# Elliptical neutron-focusing supermirrors with Ni–P-plated Al–Si alloy substrates for multi-incident-angle neutron reflectometry

**DOI:** 10.1107/S1600576726009088

**Published:** 2026-09-15

**Authors:** Yan Wei, Takuya Hosobata, Norifumi L. Yamada, Masahiro Hino, Masako Yamada, Yutaka Yamagata, Shinya Morita

**Affiliations:** a Department of Machine System Engineering, Graduate School of Advanced Science and Technology, Tokyo Denki University, Adachi-ku, Tokyo, Japan; b RIKEN Center for Advanced Photonics RIKEN Wako-shi Saitama Japan; c Institute of Materials Structure Science High Energy Accelerator Research Organization Tsukuba-shi Ibaraki Japan; d Institute for Integrated Radiation and Nuclear Science, Kyoto University, Sennan-gun, Osaka, Japan; e Institute of Materials Structure Science High Energy Accelerator Research Organization, Naka-gun Ibaraki Japan; European Spallation Source ERIC, Sweden

**Keywords:** diamond machining, polishing, focusing mirrors, neutron optics

## Abstract

This study introduces a novel approach for multi-incident-angle neutron reflectometry utilizing metallic elliptical supermirrors to enable the simultaneous focusing of two distinct neutron beams. The supermirror is composed of depth-graded NiC/Ti multilayers and functions as a reflector for grazing-incidence neutrons. Two mirror pairs consisting of precise elliptical supermirrors with diamond-machined and polished Ni–P-plated Al–Si alloy substrates were fabricated. Thermal deformation is minimized by this Al–Si alloy, which has a thermal expansion coefficient similar to that of Ni–P. The focusing performance was evaluated using the SOFIA neutron reflectometer located at BL16, Materials and Life Science Experimental Facility, Japan Proton Accelerator Research Complex. The experimental results confirmed that the two neutron beams converged to a single spot with a full width at half-maximum of 0.26 mm. Optimizing the simultaneous focusing of dual beams presents technical challenges that warrant further investigation in the future.

## Abstract 2

Multi-incident-angle neutron reflectometry is realized using elliptical metallic supermirrors for dual-beam focusing. This approach presents new opportunities for efficient interface analysis.

## 1. Introduction

Neutron reflectometry (NR) is an excellent method for studying the surfaces and interfaces of thin films in biology and materials science. Specular neutron reflectivity is measured as a function of momentum transfer *q*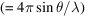, where θ is the incident angle to the sample and λ is the neutron wavelength. This measurement provides information on the scattering length density profiles as a function of depth from the surface, enabling evaluation of the thickness, roughness, composition and other structural parameters of the layers with sub-nanometric spatial resolution. Neutrons have several advantages over X-rays: they are more sensitive to light elements and isotopes, have greater penetration depths, and respond to magnetic properties. Consequently, NR has emerged as a powerful tool for investigating the surface behaviors of materials (Russell, 1990 ▸). For example, neutrons exhibit deep penetration capabilities and are nondestructive, making them ideal for *in situ* experiments and characterization of the surface and interface microstructures of polymer-bonded explosives composed of light elements (Bai *et al.*, 2023 ▸). Such *in situ* NR studies are widely used to observe metallic lithium plating and investigate electrochemical interfaces involving Li^+^-containing electrolytes (Avdeev *et al.*, 2017 ▸; Hirayama *et al.*, 2010 ▸; Browning *et al.*, 2014 ▸). *Operando* studies on the charging and discharging processes of lithium-ion batteries have also been performed using NR (Kawaura *et al.*, 2020 ▸).

The flux of neutron sources is considerably lower than that of synchrotron X-rays, necessitating longer exposure times to collect data with good statistics. Consequently, the time resolution of NR for observing structural changes in transient processes is typically limited to the order of seconds (Yamada *et al.*, 2015 ▸), whereas synchrotron X-rays can reach a resolution of tens of milliseconds (Schumi-Mareček *et al.*, 2024 ▸). Although achieving such a time resolution with neutrons remains challenging, elliptical neutron mirrors have been proposed to focus diverging neutrons, thereby enhancing the neutron flux at the sample position (Hosobata *et al.*, 2017 ▸; Hosobata *et al.*, 2019 ▸; Hussey *et al.*, 2018 ▸; Mühlbauer *et al.*, 2008 ▸; Takeda *et al.*, 2016 ▸; Torikai *et al.*, 2011 ▸; Stahn & Glavic, 2016 ▸; Wu *et al.*, 2019 ▸; Yang *et al.*, 2021 ▸). Through this design, neutrons diverging from a point source at one focal point are reflected by the supermirror coating and converge at a second focal point with large beam divergence. A neutron supermirror coating is a depth-graded multilayer reflector designed to reflect neutrons at grazing angles up to *m* times the critical angle of total reflection for natural nickel (Akiyoshi *et al.*, 1992 ▸; Hino *et al.*, 2015 ▸). This enhancement factor is expressed by the *m* value. A Montel mirror system with four focusing mirrors was implemented at the Amor reflectometer at the Paul Scherrer Institute, achieving a full beam width of 0.8 mm at the sample position (Stahn & Glavic, 2016 ▸). Meanwhile, we have developed a one-dimensional focusing mirror for illuminating small samples (Hosobata *et al.*, 2017 ▸; Hosobata *et al.*, 2019 ▸; Takeda *et al.*, 2016 ▸), achieving a minimum beam size of 0.3 mm at full beam width.

When analyzing thin films through specular NR, a wide *q* range can be measured by two primary approaches. One is a monochromatic θ–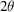 scan, in which a neutron beam with a fixed wavelength is used and the incident angle is varied while adjusting the detector angle to satisfy the specular reflection condition (van Well *et al.*, 2000 ▸; Cousin & Fadda, 2020 ▸). The other is time-of-flight NR (TOF-NR), in which a pulsed neutron beam with a broad wavelength band is used to obtain a range of *q* values at a fixed incident angle (Cousin & Fadda, 2020 ▸). In TOF-NR, achieving a sufficiently wide *q* range generally requires measurements at multiple incident angles. To further improve NR efficiency, we previously proposed a method based on multi-incident-angle NR (MI-NR) (Yamada *et al.*, 2020 ▸) to expand the accessible *q* region in a single measurement. In the proposed approach, two neutron beams with different incident angles are focused simultaneously onto the sample position, enabling the measurement of a wider *q* region of layered structures and improving measurement efficiency. However, fabricating such a system remains challenging because of the stringent requirements related to the accuracy of the optical elements and mechanical precision of their alignment.

Ni–P-plated metal substrates are uniquely suited for neutron-focusing supermirrors because of their stability and form accuracy. In addition to Ni–P, glass and silicon are commonly used as substrate materials. Although float glass and chemo-mechanically polished silicon wafers are cost-effective options and have been employed to develop functional focusing devices in various forms (Torikai *et al.*, 2011 ▸; Wu *et al.*, 2019 ▸; Yang *et al.*, 2021 ▸), their form accuracy is limited when they are shaped through mechanical bending. The ultra-precise forming of bulk glass through a local wet etching technique offers a method to overcome this limitation (Yamamura *et al.*, 2009 ▸). However, the initial rough shaping of glass involves substantial material removal by grinding, making this a time-consuming process. Further, neutron guides are extended as close as possible to the neutron source to maximize the neutron flux delivered to the instruments. Glass substrates lack stability against irradiation, temperature variations and mechanical stress, restricting their installation to positions beyond 1.5 m from the moderator. Consequently, metal substrate supermirrors are widely used in neutron optical systems, including neutron guides that transport neutrons from the source to the instruments (Schanzer *et al.*, 2010 ▸; Khaykovich *et al.*, 2020 ▸). Additionally, Ni–P plating provides an efficient and convenient solution for preforming, because N–P can be deposited onto substrates that are rapidly preformed by milling (Hosobata *et al.*, 2017 ▸; Hosobata *et al.*, 2019 ▸; Takeda *et al.*, 2016 ▸). The machinability of Ni–P-plated metal substrates makes them an ideal choice for use in supermirrors within neutron optical assemblies, wherein multiple neutron-focusing supermirrors are mechanically aligned. One advantage of such an assembly is its ability to achieve larger mirror areas beyond the limitations of the production equipment. This concept has been successfully demonstrated through the realization of an elliptical focusing mirror for a neutron reflectometer (Hosobata *et al.*, 2019 ▸) and ellipsoidal mirrors for a neutron resonance spin-echo spectrometer (Hosobata *et al.*, 2018 ▸). Another potential advantage of a supermirror deposited on metal substrates is the ease with which elements can be assembled through precise machining to handle multiple neutron beams with different flight paths.

In this study, we present a neutron optical system for MI-NR that demonstrates the feasibility of simultaneously focusing two discrete neutron beams. The system employs four elliptical neutron-focusing supermirrors, which utilize a novel fabrication approach involving an Al–Si alloy as the bulk material beneath the Ni–P plating. The similar thermal expansion coefficients of the Al–Si alloy and Ni–P plating enhance the thermal stability of the neutron supermirrors.

The remainder of this paper is organized as follows: First, we outline the motivation behind developing this neutron-focusing system—specifically its application in MI-NR (Yamada *et al.*, 2020 ▸)—and describe the geometry of the system. This proposed system aims to enhance the performance of SOFIA, a horizontal-type neutron reflectometer installed at BL16 in the Materials and Life Science Experimental Facility (MLF) at the Japan Proton Accelerator Research Complex (J-PARC) (Mitamura *et al.*, 2013 ▸; Yamada *et al.*, 2011 ▸). Neutron-focusing supermirrors have been integrated into the SOFIA reflectometer to facilitate the illumination of small samples (Hosobata *et al.*, 2017 ▸; Hosobata *et al.*, 2019 ▸; Takeda *et al.*, 2016 ▸). We provide detailed information on the design and fabrication of the four elliptical supermirrors used in the system. Finally, we present the results of substrate fabrication and neutron-focusing experiments. Although the results indicate a need for further improvements in alignment precision, they demonstrate the feasibility of a focusing system that can handle multiple neutron beams.

## 2. Neutron-focusing geometry and application

In the MI-NR configuration for the SOFIA reflectometer, two discrete neutron beams illuminate the sample simultaneously (Yamada *et al.*, 2020 ▸). By leveraging the semi-white beam characteristic of the SOFIA reflectometer, the wavelength spectrum of 0.25–0.9 nm (extendable to 1.84 nm) enables reflectivity measurements across a wide *q* region, even in conventional single beam modes.

However, reflectivity data collected at different incident angles are required to observe interference from layers of various thicknesses (from a few to hundreds of nanometres) across the entire measurable *q* region. The simultaneous measurement of reflectivity at different incident angles via MI-NR optics enables us to measure the entire observable *q* region without any angle scan. This feature is especially useful for time-sliced measurements, which enable the observation of structural transitions, such as those during the charging–discharging cycles of batteries.

In addition to generating two neutron beams with different incident angles, the proposed system employs a double-reflection configuration for each beam path. If a single mirror was used to achieve the required angular separation, a high-*m* supermirror (*e.g.*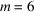 instead of 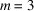) would be necessary, significantly reducing the reflectivity. An angular difference of 2.32° between the two incident beams is achieved by using two reflections with 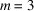 supermirrors in each beam path, thereby avoiding the intensity loss associated with a single 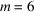 supermirror. A more detailed discussion of the design concept is provided in our previous paper (Yamada *et al.*, 2020 ▸).

**Figure 1:**
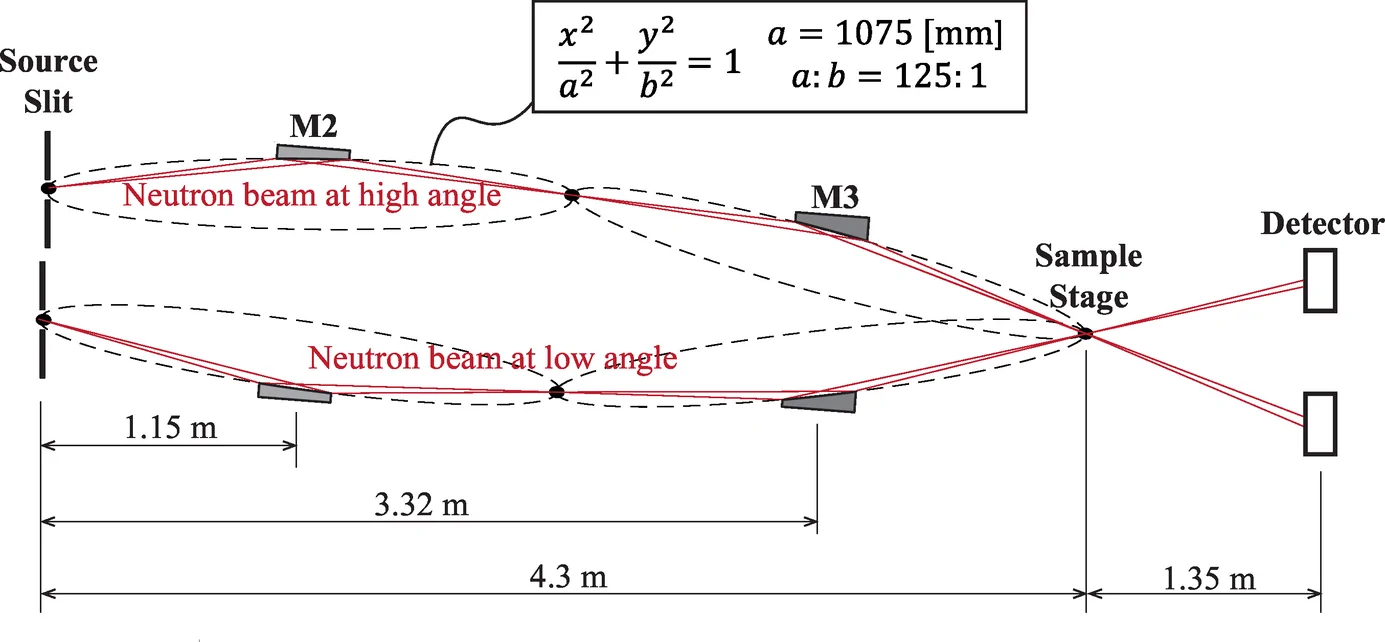
Neutron-focusing geometry of MI-NR for SOFIA. Two discrete neutron beams originating from separate source slits are focused on the sample stage at different angles. This convergence is achieved through double reflections using elliptical neutron-focusing mirror pairs M2 and M3.

As illustrated in Fig. 1 ▸, two slits serve as virtual neutron sources from which the beams diverge. These beams are directed toward the sample position using two elliptical neutron-focusing mirror pairs (M2 and M3) located at distances of 1.15 and 3.32 m from the source slits, respectively. Each mirror pair consists of top and bottom mirrors that reflect the high- and low-angle beams, respectively. The mirrors have an effective length of 300 mm. The sample is positioned 4.3 m away from the source slits, where the two beams are designed to intersect and focus. The slit widths can be controlled individually such that the two beams with different incident angles illuminate the same area on the sample surface. Although the positions of the slits, two mirror pairs and sample stage can be adjusted using stepping motors for optical alignment, the two mirrors within each pair are fixed relative to each other during assembly and cannot be adjusted independently. Consequently, achieving precise mechanical alignment of the relative angle within each mirror pair during assembly is crucial.

## 3. Design and fabrication of the neutron-focusing supermirrors

**Figure 2:**
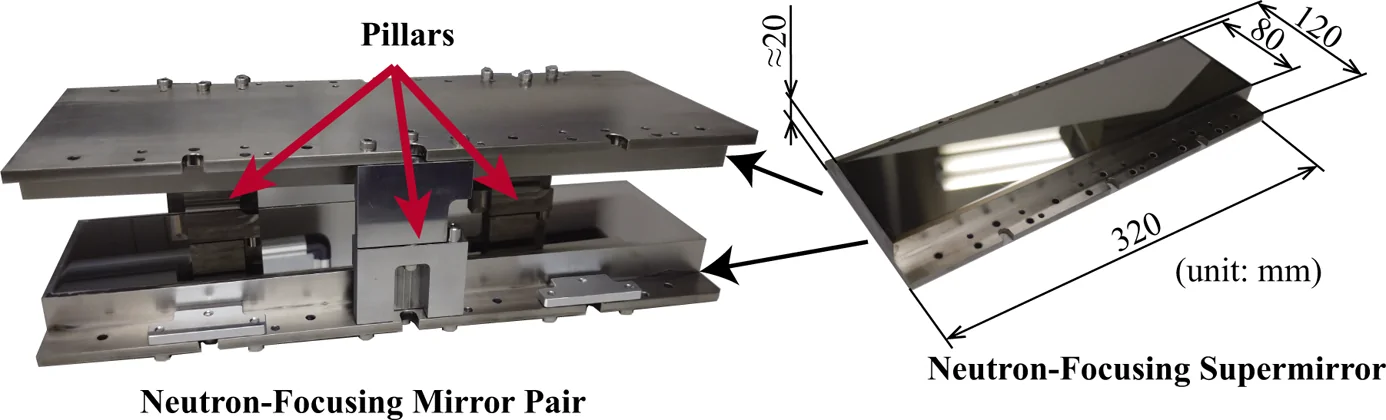
Photograph of a mirror pair fabricated using Ni–P-plated substrates. The mirror pair includes two neutron-focusing supermirrors, positioned on the top and bottom, supported by three pillars.

Fig. 2 ▸ shows a photograph of a mirror pair fabricated using Ni–P-plated substrates. The mirror pair is fixed using three pillars rather than individual adjustment mechanisms for each mirror to ensure a stable relative angle between mirrors. This design simplifies the mechanical structure while maintaining a well-defined relative mirror angle.

Each mirror is fabricated as follows: Initially, a roughly premachined aluminium block is plated with Ni–P. The mirror surface is then shaped into an ellipse via diamond cutting. Subsequently, the mirrors undergo a small-tool polishing procedure using alumina and colloidal silica slurries to achieve sub-nanometre surface roughness. Finally, an ion beam sputtering technique is employed to deposit a supermirror multilayer coating onto the mirror surface. These fabrication methods have previously been established and documented in our published papers (Hosobata *et al.*, 2017 ▸; Hosobata *et al.*, 2019 ▸; Takeda *et al.*, 2016 ▸).

The current study introduces two new advancements in addition to these established techniques. The first involves utilizing a new material beneath the plating, while the second technique relates to the alignment method implemented through the three pillars.

We employ an Al–Si alloy (40% Si) as the base substrate, replacing the Al–Mg alloy (2.5% Mg) used in our previous studies. When fabricating neutron optical devices, material selection is constrained by the need to minimize radioactivation. Although Al–Mg is typically selected because of its suitability, it presents a challenge when combined with Ni–P plating because of the difference in thermal expansion coefficients, which leads to temperature-dependent deformations. The Al–Mg alloy exhibits a linear expansion coefficient of approximately 23.8 × 10^−6^ K^−1^, which is almost twice that of Ni–P, measuring 11.5 × 10^−6^ K^−1^. This difference induces bimetallic deformation when the thickness of the Ni–P varies between the mirror surface and back side, often occurring during the diamond-cutting process. Consequently, to suppress deformation caused by the thermal expansion mismatch between Al–Mg and Ni–P, strict temperature control is required during fabrication and installation in the neutron beamline. The Al–Si alloy was selected to reduce this thermal expansion mismatch while maintaining suitability for neutron optical applications.

The Al–Si alloy employed in this study (RSA 443, manufactured by RSP Technology) has a thermal expansion coefficient of 13.5 × 10^−6^ K^−1^, which is close to that of Ni–P. Past research has validated the effectiveness of combining the Al–Si alloy with Ni–P in minimizing thermal deformation for optics, even under cryogenic conditions (Rohloff *et al.*, 2010 ▸). This suggests that the thermal stability of our supermirror substrates will also be enhanced. Additionally, both Al and Si exhibit neutron transparency and they do not produce long-life radioactive isotopes upon neutron exposure. These characteristics suggest improved thermal stability and radiation resistance, making Ni–P-plated Al–Si alloy substrates an excellent choice for neutron-focusing mirrors.

**Figure 3:**
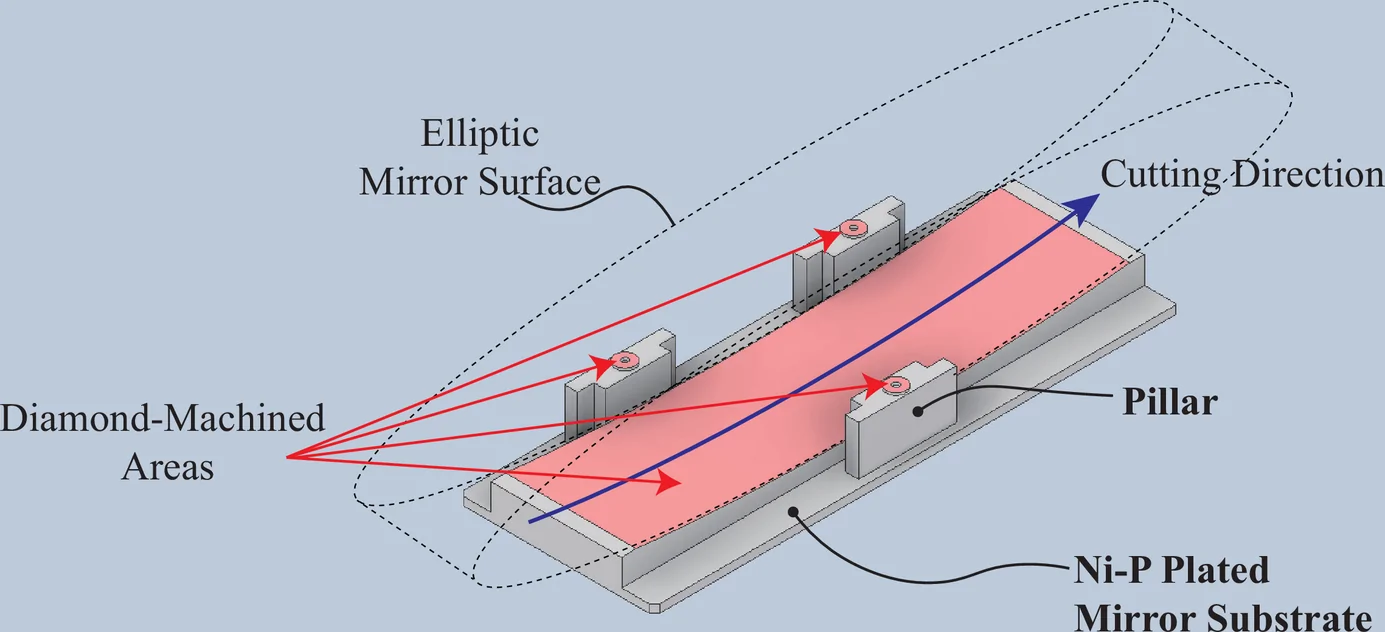
Diamond-machined areas of the supermirror substrate, including the elliptical mirror surface and support points on the three pillars.

As shown in Fig. 3 ▸, the heights of the three pillars are adjusted via diamond cutting along with the processing of the mirror surface to achieve precise alignment during the assembly of the mirror pairs.

## 4. Experimental results

### 4.1. Thermal deformation measurement

A comparative experiment using Ni–P-plated Al–Si and Al–Mg alloy substrates was conducted to investigate their thermal deformations at various temperatures. Prior to the experiment, the two alloys were milled to an identical form. The dimensions are given in Fig. 2 ▸. The substrates had a nonuniform thickness ranging from 20.3 to 21.5 mm, because one face is curved to create an elliptical shape with a semi-major axis of 1075 mm and semi-minor axis of 8.6 mm. An Ni–P-plating layer with a thickness of 0.1 mm was applied to all surfaces, and the elliptical face underwent a diamond-cutting process to remove approximately 0.05 mm from its surface. Consequently, a difference in plating thickness was produced. This difference causes asymmetry between the mirror and back sides of the substrates, leading to bimetallic deformation.

**Figure 4:**
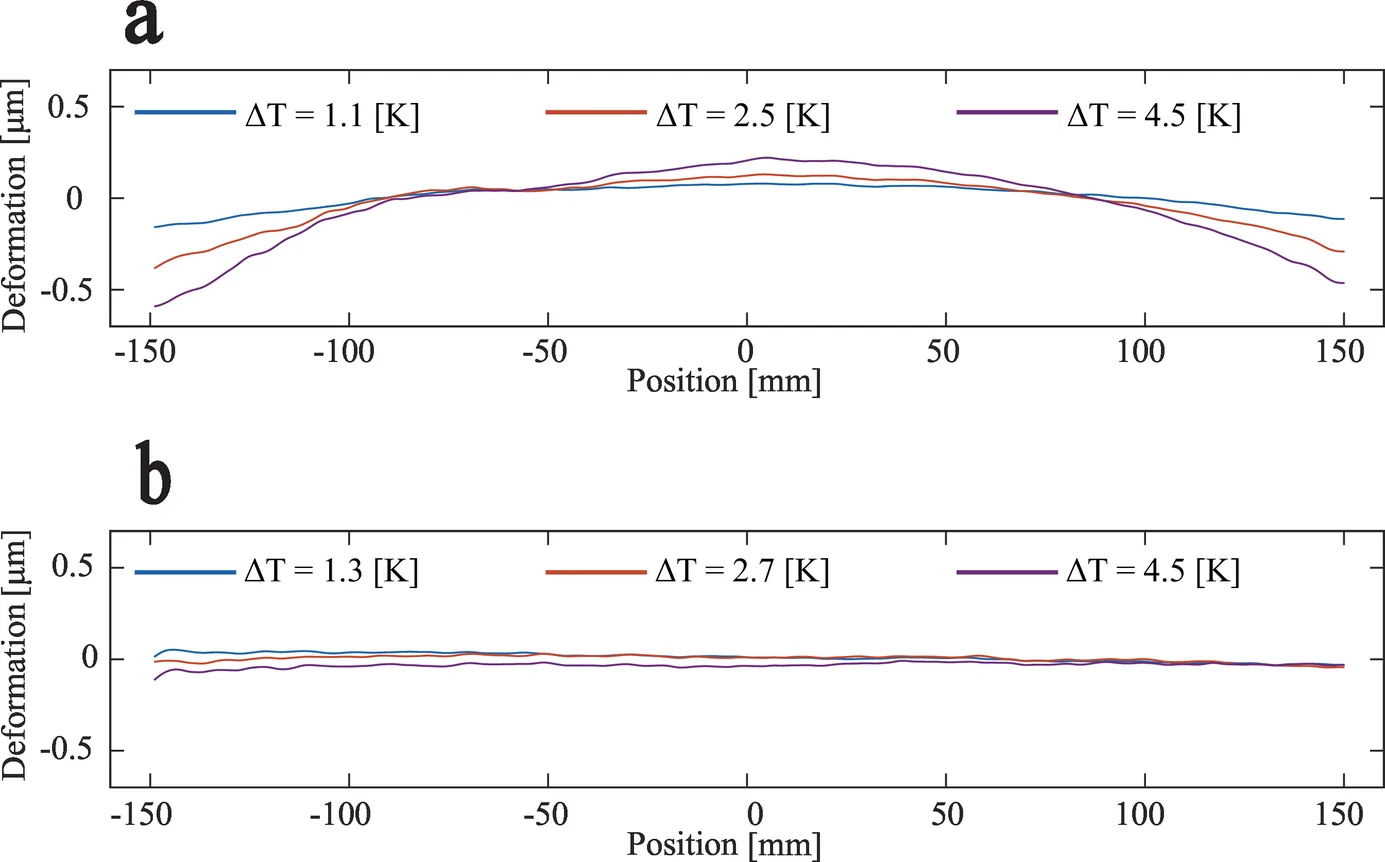
Results of the thermal expansion experiment. (*a*) Thermal deformation of the Ni–P-plated Al–Mg alloy mirror substrate. (*b*) Thermal deformation of the Ni–P-plated Al–Si alloy mirror substrate.

The experiment was conducted in a temperature-controlled room stabilized at approximately 20 °C. Heated air was applied to the substrate to induce temperature changes. The measuring machine was enclosed using a custom-made temperature-control system to stabilize air conditions and maintain the desired temperature range. Temperature monitoring was conducted using three K-type thermocouple sensors placed on the substrate and one placed in the surrounding air. The shape of the substrate was measured using a 3D coordinate measuring machine (LEGEX, Mitutoyo Corporation) equipped with a laser auto-focus probe (PFU-3, Mitaka Kohki Corporation). Initially, the form of the substrate was measured at a temperature of approximately 20 °C. Subsequently, the form was remeasured at different temperatures. The resulting deformation from the original temperature is shown in Fig. 4 ▸. During this measurement, the substrates were positioned on top of three ball-tip pins to ensure static support.

A comparison of the two mirror substrates revealed that the Al–Mg-based substrate exhibited a significant warp of approximately 0.81 µm with a corresponding temperature rise of 4.5 °C. This large deformation is sufficient to result in observable shifts in focal points. By contrast, the deformations observed in the Al–Si-based substrate were 0.08, 0.07 and 0.10 µm for temperature increases of 1.3, 2.7 and 4.5 °, respectively. The small difference between the first two measurements is within the measurement uncertainty. Therefore, no clear proportional trend between deformation and temperature rise was identified within the investigated temperature range. The results suggest that the Al–Si-based substrate exhibits superior thermal stability compared with the Al–Mg-based substrate, making it more favorable for neutron-focusing supermirrors.

### 4.2. Form and assembly accuracy

The substrate forms were measured using the same machine as used for the thermal deformation experiment under two conditions: as-polished and after applying a NiC/Ti multilayer supermirror coating with a thickness of 3 µm. These coatings deposited via ion-beam sputtering (Hino *et al.*, 2015 ▸) provide neutron reflection with a critical angle three times higher than that of pure nickel. Measuring the form before and after coating is crucial because the coating-induced membrane stress deforms the substrates and affects the focusing performance of the mirror.

**Figure 5:**
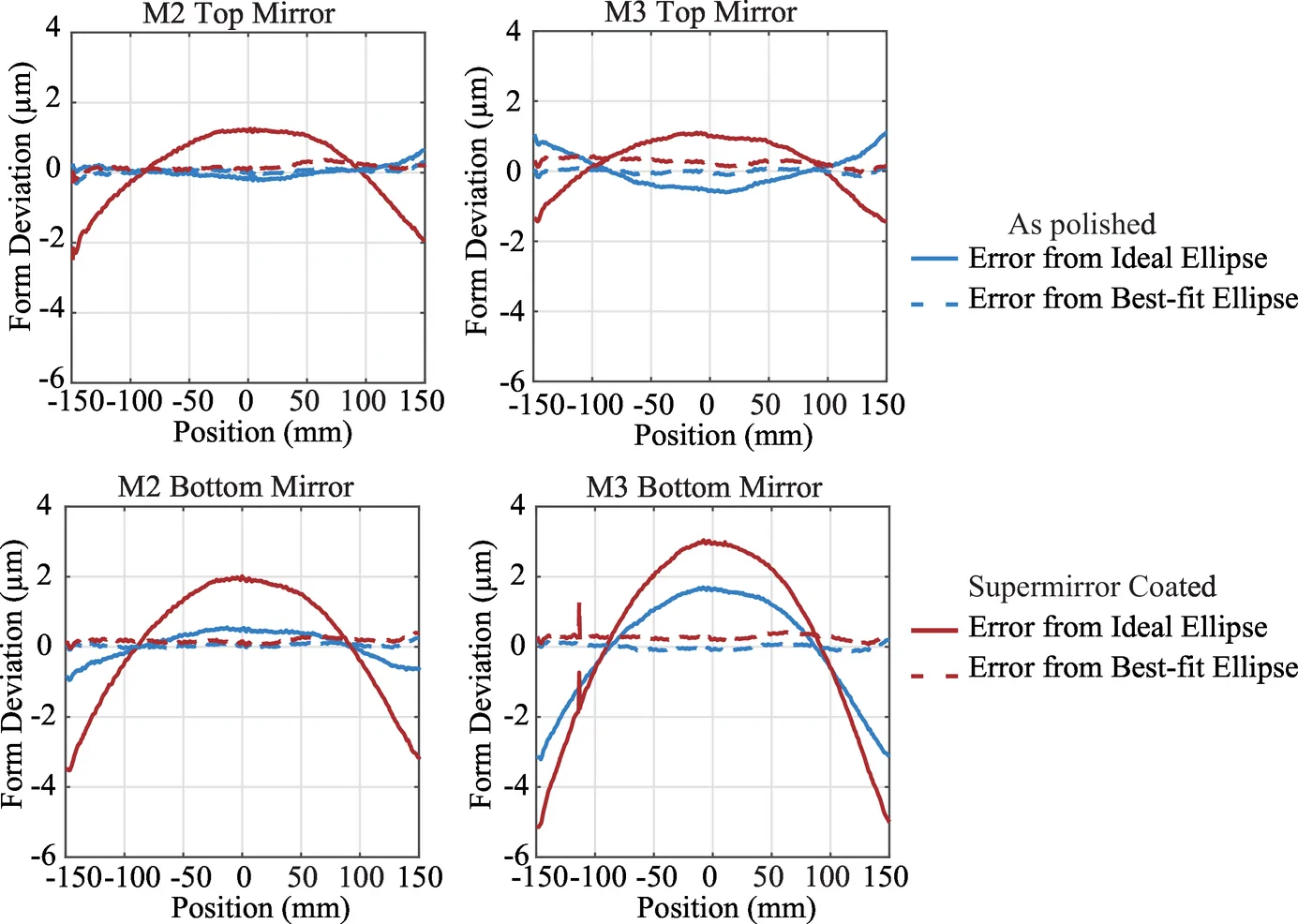
Form deviations of neutron-focusing mirrors in the as-polished and supermirror-coated states relative to the designed and best-fit ellipses. Measurements were performed using a 3D coordinate measuring machine (LEGEX, Mitutoyo Corporation) equipped with a laser auto-focus probe (PFU-3, Mitaka Kohki Corporation).

Fig. 5 ▸ displays the form deviations of the mirrors relative to the designed and best-fit ellipses, where the semi-major axis was used as the fitting parameter.

Form deviations of up to 6 µm from the designed ellipses were observed prior to the supermirror coating. These deviations were attributed to substrate deformations arising from how the substrates were fixed during the ultra-precision machining process, as well as to the residual stress released when removing the Ni–P plating in the diamond-cutting process. The M3 bottom mirror exhibited an abrupt dis­place­ment at approximately −120 mm because of a tiny adhesion on the surface during measurement. After coating, the form deviation increased by 3 to 5 µm; however, the apparent deformation became smaller when the error was evaluated with respect to the best-fit ellipses. This implies that the measured profiles followed an elliptical shape, although the semi-major axes differed from the design values. The semi-major axes of the fitted ellipses were 1096 and 1112 mm for the M2 top and bottom mirrors and 1091 and 1132 mm for the M3 top and bottom mirrors, respectively. The deformation after coating exhibited good repeatability among the four mirrors, suggesting that deformation can be predicted and that precompensation machining can ensure the figure accuracy of the coated surfaces. This discrepancy contributes to the suboptimal performance of the proposed neutron-focusing system, as indicated by the neutron-focusing experiment results described below.

**Figure 6:**
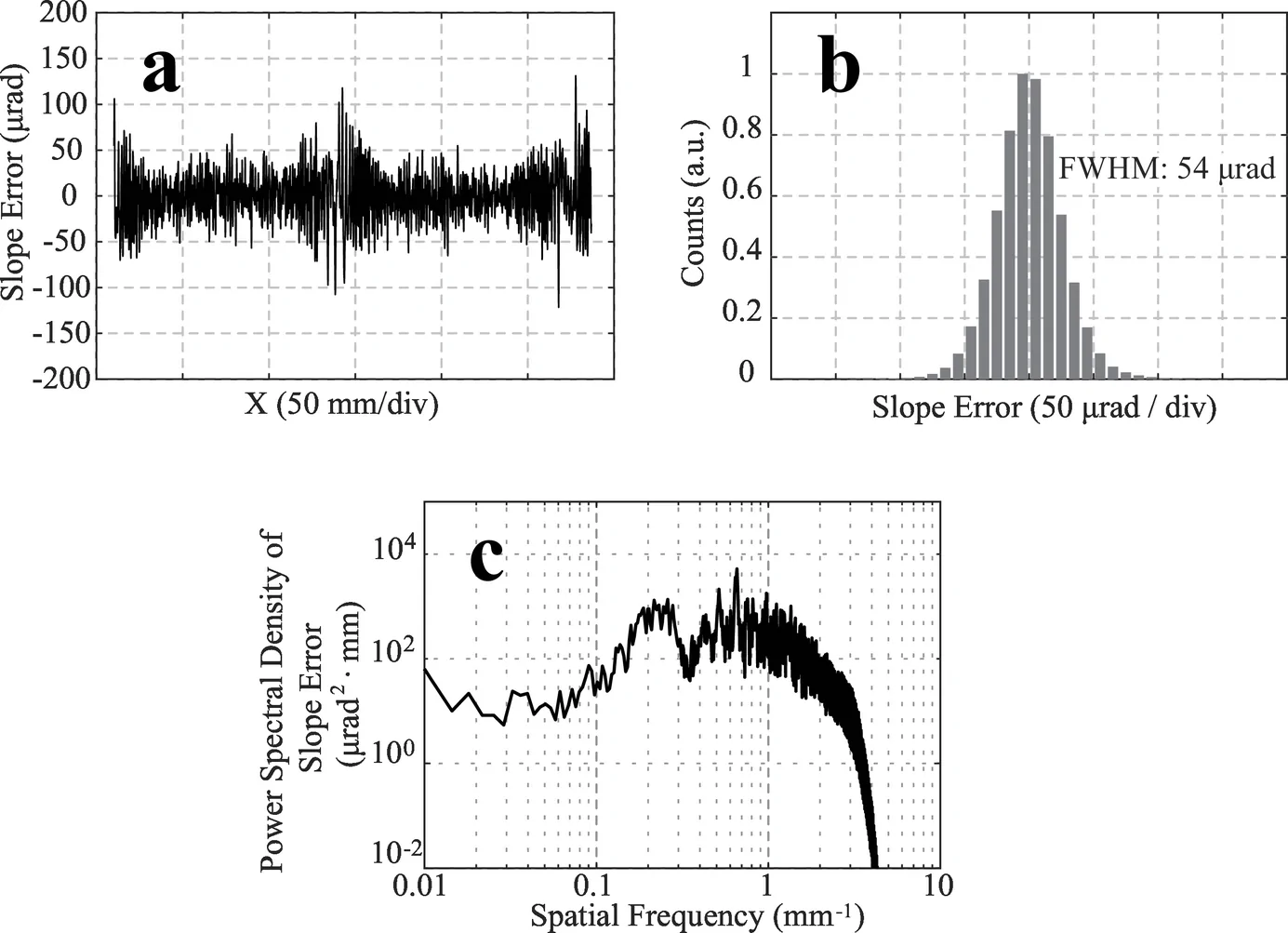
Slope error caused by waviness on one of the elliptical supermirrors composing the dual-beam neutron-focusing system: (*a*) spatial distribution of the slope error along the center line of the mirror, (*b*) its histogram and (*c*) its power spectral density calculated from the form measurement using a laser interferometer (Verifire QPZ, Zygo Corporation).

Not only the macroscopic form deviations but also the slope error resulting from surface waviness must be considered for evaluating the focusing performance of the mirror. The slope error distribution with respect to the best-fit ellipses on one of the mirrors (specifically, the top mirror of the M2 pair) was analyzed using a laser interferometer (Verifire QPZ, Zygo Corporation), and the results are illustrated in Fig. 6 ▸.

The measured slope error distribution exhibited a full width at half-maximum (FWHM) of approximately 54 µrad, corresponding to a blur of 0.12 mm in the focal spot. The power spectral density of the slope error revealed peaks at spatial frequencies of 0.52 and 0.66 mm^−1^. On investigating the cause of this waviness, a calculation error was identified in the tool path generation. We had unintentionally rounded the tool path to the nearest 100 nm; this rounding error was transmitted to the motion of the ultra-precision machine tool. We aim to rectify this error in our future work.

After the assembly, the relative tilt angle of each mirror pair was measured using a touch probe on the previously described 3D coordinate measurement machine. The tilt angle of the mirror pair was measured with the mirror assembly laid horizontally because the probe could only be inserted from above. In this configuration, the probe scanned the mirror surface from the upper side to determine the tilt angle. Comprehensive information related to the alignment precision of each mirror pair was obtained by combining the data obtained from this measurement with the form measurement data of each mirror. The tilt angle errors for the M2 and M3 mirror pairs were determined to be 32 and −84 µrad, respectively. These angles indicate the tilt of the mirror on the top side when the bottom mirror was adjusted to the desired geometry. Notably, the three-pillar structure serves as a mechanical alignment method for defining the relative angle between the top and bottom mirrors during assembly, although small additional deformation caused by local contact forces, assembly stress or gravitational loading cannot be completely excluded. In the present study, each mirror was measured individually under a simulated three-point support condition. Therefore, the measured form deviations include possible support-induced deformation under this measurement condition. However, deformation induced specifically by the final three-pillar assembly could not be quantitatively separated from the intrinsic form and alignment errors of each mirror, because the form of the mirror pair in the final assembled state could not be measured directly. This effect will be further evaluated in future work by *in situ* form measurement of the assembled mirror pair or by numerical deformation analysis.

### 4.3. Neutron-focusing performance test

Completed neutron-focusing supermirror pairs were installed in the SOFIA neutron reflectometer to assess their focusing performance. For this experiment, both source slits had an opening width of 0.05 mm. The neutron intensity distribution at the sample position was determined by scanning a cadmium (Cd) slit with a 5 µm opening and measuring the transmitted neutron counts using a ^6^LiF/ZnS scintillation counter.

**Figure 7:**
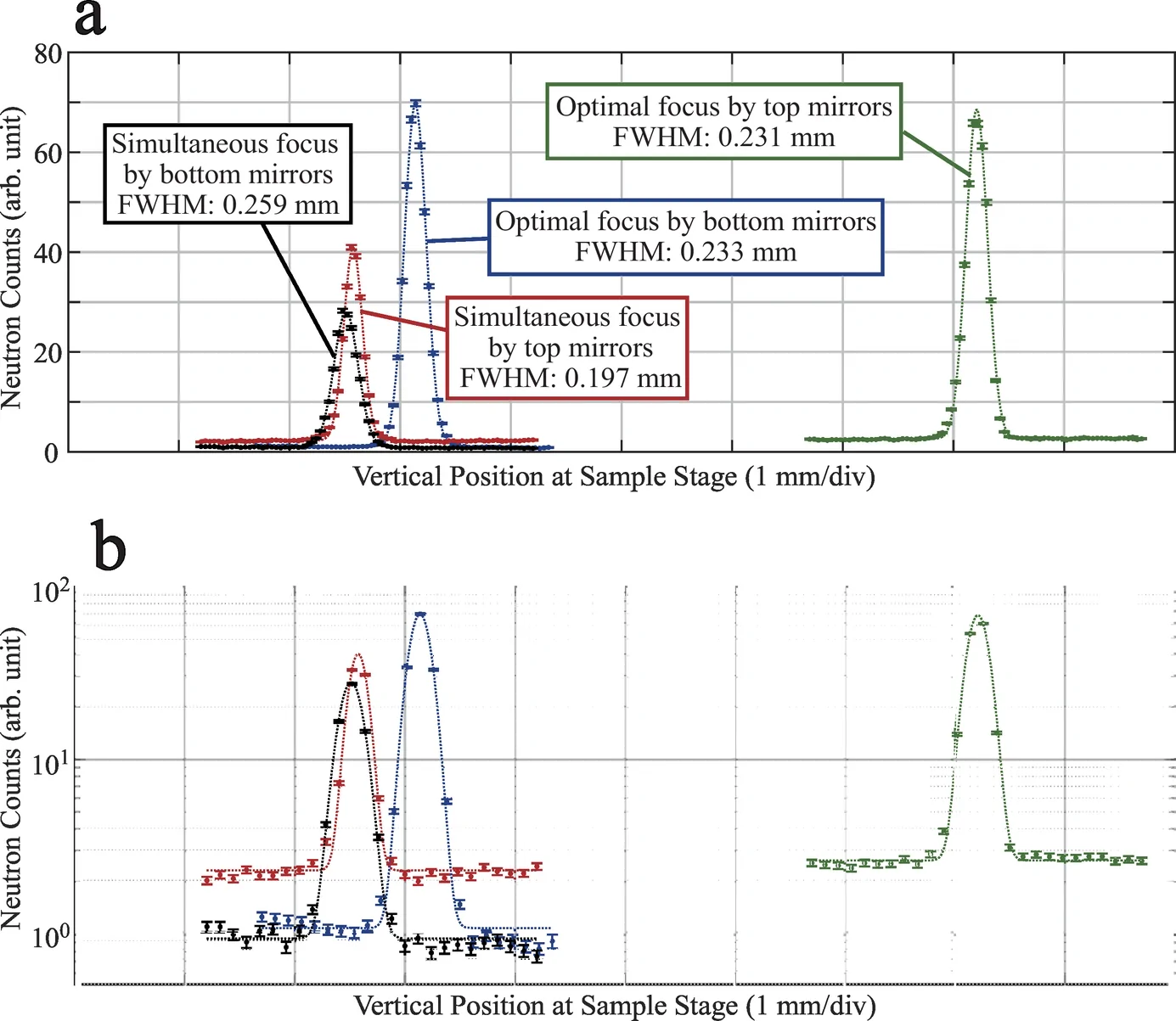
Profiles of neutron beams focused by the dual-beam neutron-focusing system comprising two pairs of elliptical neutron-focusing supermirrors. Independently optimized profiles are shown in blue for the bottom mirrors and green for the top mirrors; simultaneous-focusing profiles are shown in black for the bottom mirrors and red for the top mirrors. Dotted lines represent the best-fit Gaussian curves. (*a*) Linear scale and (*b*) logarithmic scale.

Fig. 7 ▸ displays the measured profiles of the focal spots from the neutron-focusing mirrors. The blue and green markers represent the focal spots obtained by independently aligning the bottom and top mirrors, respectively, to achieve the sharpest spots. However, the optimal tilt and height of the mirrors were different between beam paths, indicating that the optimal alignment condition differs between the two beam paths. Consequently, we attempted to find a condition where the two beams converged at the same position with the optimal mirror alignment for the lower beam. We identified this condition by adjusting the positions of the virtual sources and the tilt of the downstream mirror pair (M3), as indicated by the red and black markers. These results confirmed that the two discrete neutron beams for the high and low angles successfully focused at the same position, with FWHMs of 0.197 and 0.259 mm, respectively. In the logarithmic-scale intensity profile, no pronounced long tail was observed within the dynamic range of the present measurement. The off-peak background in Fig. 7 ▸(*b*) is considered to be mainly attributable to stray neutrons counted during the focal-spot scanning measurement. In this measurement, the 5 µm-wide Cd slit was scanned at the sample position to determine the intensity distribution. By contrast, the slit located 200 mm upstream of the sample position, which is usually used to define the illuminated sample area in NR measurements, was moved downward out of the beam path during beam alignment such that it did not cut the beam. Therefore, stray neutrons outside the intended beam path could have contributed to the measured off-peak background. This background contribution is expected to be reduced in actual NR measurements when the upstream slit is inserted into the beam path.

In our previous study (Yamada *et al.*, 2020 ▸), under the reflectometry measurement geometry, a gain factor of approximately 2 was achieved with a focal spot size of 0.13 mm (FWHM). The gain factor increased with a smaller focal spot size; therefore, the gain factor of the present system was expected to be lower because of the larger focal spot size observed in this experiment.

Further optical tuning revealed that, although the two neutron beams could achieve higher intensity if we adjusted the heights and angles of the mirror pairs, a single alignment condition could not simultaneously optimize both beam paths, resulting in suboptimal focal spots during simultaneous focusing. The green and blue markers represent optimal focal spots for the top and bottom mirrors, respectively. These optimal focal spots appeared 5 mm apart and exhibited almost twice the intensity compared with that in the case of simultaneous focusing. Although the proposed dual-beam focusing system successfully achieved simultaneous focusing, it did not achieve simultaneous optimization, resulting in suboptimal performance.

The shift between the optimal spots of the top and bottom mirrors indicates two issues: (i) further adjustments to the tilt angle of each mirror pair assembly are required, and (ii) the semi-major axes of the mirrors need to match the design values more closely.

## 5. Discussion

We have developed a prototype neutron-focusing system that can handle multiple discrete neutron beams. The main components of this system are metal-substrate-based neutron supermirrors with elliptical surfaces, which provide a large reflective area for efficient neutron focusing and increased flux at the sample position while maintaining high form accuracy. We successfully focused two neutron beams with different incident angles at a single focal spot, precisely at the same sample position.

However, the limited intensity obtained during simultaneous focusing of the two beams revealed the need for further optimization. In this study, a focusing requirement of 0.1 mm was set, based on the footprint projection of a 10 mm square sample under a 10 mrad incident angle to enable the practical implementation of this system in multi-incident-angle NR. Consequently, two optimal focal spots, each with an FWHM of 0.1 mm and aligned at the same position, must be realized. This limitation can be attributed to the machining and alignment accuracy of the mirrors. Further improvements in fabrication precision are expected to enhance focusing performance and measurement efficiency.

To achieve this goal, one approach would be to enhance the absolute precision of mirror substrates in relation to the designed ellipses. Elliptical focusing mirrors can tolerate quadratic deformations when used individually because such deformations correspond to changes in the semi-major axis length, which can be compensated for through optical alignment. However, in optical assemblies such as the proposed dual-beam focusing system, such deformations become undesirable. The introduction of the Al–Si alloy in this study reduced thermal deformation during manufacturing. However, challenges related to deformation arising from fixturing and membrane stress caused by supermirror coating must be addressed. The former can be tackled by devising methods to fix the substrates with minimal stress while maintaining adequate stiffness, and the latter can be improved through the empirical prediction of deformation and subsequent corrective diamond machining based on these predictions.

The difference in beam positions between the two beam paths indicates that the relative tilts of the mirror pairs require further adjustments. In the present design, each mirror pair was fixed using three pillars instead of employing individual adjustment mechanisms for each mirror. This configuration was adopted intentionally to simplify the mechanical structure and maintain a stable relative angle between the mirrors. However, the relative alignment between the mirrors within each pair is determined during assembly and cannot be readily evaluated or verified afterwards. The experimental results revealed that the neutron beam paths differ from the initially assumed trajectories, suggesting that the internal alignment condition may not be fully consistent with the design assumptions. On the basis of the experimentally observed beam paths, future improvements could focus on developing methods to evaluate and control the relative alignment of the mirrors with higher accuracy.

## 6. Conclusion

This study has proposed a dual-beam focusing system for NR that included two pairs of elliptical neutron-focusing supermirrors. Further, the feasibility of simultaneously focusing two neutron beams for MI-NR was demonstrated. The supermirrors utilized Ni–P-plated substrates with an Al–Si alloy as the base metal to prevent bimetallic deformation during fabrication. Each mirror pair was precisely aligned using three pillars, whose heights were adjusted by diamond cutting during the elliptical surface forming process. The assembled focusing system was installed successfully in a neutron reflectometer, demonstrating the simultaneous focusing of two discrete beams at the designated sample position. Although simultaneous optimization of the two beam paths remains a future challenge, partly because the relative alignment of the assembled mirrors cannot yet be evaluated with sufficient accuracy, the present results support the feasibility of multi-beam neutron-focusing optics using Ni–P-plated mirror substrates.

## Data Availability

The data that support the findings of this study are available from the corresponding author upon reasonable request.
